# The role of the dorsoanterior striatum in implicit motivation: the case of the need for power

**DOI:** 10.3389/fnhum.2013.00141

**Published:** 2013-04-19

**Authors:** Oliver C. Schultheiss, Anja Schiepe-Tiska

**Affiliations:** ^1^Department of Psychology, Friedrich-Alexander UniversityErlangen, Germany; ^2^Centre for International Student Assessment (ZIB), TUM School of EducationTechnische Universität München, Germany

**Keywords:** implicit motives, personality, reinforcement, learning, dopamine, power motivation, striatum, caudate nucleus

## Abstract

Implicit motives like the need for power (nPower) scale affective responses to need-specific rewards or punishments and thereby influence activity in motivational-brain structures. In this paper, we review evidence specifically supporting a role of the striatum in nPower. Individual differences in nPower predict (1) enhanced implicit learning accuracy, but not speed, on serial-response tasks that are reinforced by power-related incentives (e.g., winning or losing a contest; dominant or submissive emotional expressions) in behavioral studies and (2) activation of the anterior caudate in response to dominant emotional expressions in brain imaging research. We interpret these findings on the basis of Hikosaka et al.'s (2002a) model of central mechanisms of motor skill learning. The model assigns a critical role to the dorsoanterior striatum in dopamine-driven learning of spatial stimulus sequences. Based on this model, we suggest that the dorsoanterior striatum is the locus of nPower-dependent reinforcement. However, given the centrality of this structure in a wide range of motivational pursuits, we also propose that activity in the dorsoanterior striatum may not only reflect individual differences in nPower, but also in other implicit motives, like the need for achievement or the need for affiliation, provided that the proper incentives for these motives are present during reinforcement learning. We discuss evidence in support of such a general role of the dorsoanterior striatum in implicit motivation.

Implicit motives represent enduring non-conscious, affect-based preferences that drive humans' behavior toward the attainment of certain types of incentives, such as those related to power/dominance, social affiliation, attachment, achievement/mastery, food, or sex that are fundamental for survival in the social and non-social world (Schultheiss and Wirth, 2008; Schultheiss and Brunstein, 2010). The need for power (nPower) is an implicit motivational disposition to experience one's own impact on others as rewarding and others' impact on oneself as aversive (Winter, 1973; Schultheiss, 2008). Research has accumulated evidence for a critical role of this need in implicit learning of behavior that is instrumental for obtaining rewards and avoiding punishers in the power domain. Other research suggests an involvement of the dorsoanterior striatum in nPower-associated responses to power incentives. In the present paper, we first briefly review these two lines of research and then integrate them into a model of nPower-dependent individual differences in instrumental learning mediated by the dorsoanterior striatum. In closing, we will discuss the role of the striatum in the context of other motivational needs.

## nPower: measurement and validity as a motivational need disposition

Measures of nPower were developed and successively fine-honed through studies in which researchers studied effects of experimentally aroused power motivation on the content of imaginative stories that research participants wrote about picture cues (Veroff, 1957; Uleman, 1972; Winter, 1973). In this way, content-coding systems for nPower were derived that have causal validity (see Borsboom et al., 2004) and that capture the themes that power-motivated people spontaneously think about and inject into picture stories. Story themes related to nPower can be objectively coded from picture stories, as documented by inter-rater reliabilities of typically >0.85 (Schultheiss and Pang, 2007). nPower scores derived from content-coding have good retest reliability (Schultheiss and Pang, 2007) and are particularly suitable for predicting spontaneous behavior in response to non-verbal incentives, long-term behavioral trends, and health outcomes such as immune system functioning and cardiovascular disease (McClelland, 1989; Schultheiss, 2008; Fodor, 2010). Notably, nPower is considered to be an implicit motive, because content-coded nPower does not generally correlate with questionnaire measures of self-attributed (i.e., explicit) power motivation, dominance, or aggression (e.g., Pang and Schultheiss, 2005; Stanton and Schultheiss, 2007), and neither do these explicit measures account for the motivational outcomes and phenomena that nPower predicts (for reviews of the differences between implicit and explicit motive measures, see McClelland et al., 1989; Schultheiss, 2008; Stanton et al., 2010).

Like other implicit motives (e.g., the needs for achievement, affiliation, or intimacy), nPower determines the degree to which a person finds pleasure in, or *likes* (cf. Berridge, 2003), a particular class of rewards, which, in the case of nPower, consist of episodes in which the person has impact on others or dominates others. It also determines the degree to which a person experiences as aversive, or dislikes, a particular class of punishments, such as failing to have impact on others or being the object of others' dominance. Individual differences in nPower thus correspond to individual differences in the reward and punishment value of such episodes and, as a consequence, in the intensity and frequency with which a person strives for them or *wants* them in the case of power-specific rewards or wants to avoid them in the case of power-specific punishments.

Evidence for differential pleasure responses to dominance success or failure come from studies using objective indicators of affect as represented in facial expressions. Assessing activity of the corrugator muscle, which is involved in frowning and assumed to reflect hedonic responses to objects and events (Larsen et al., 2003), Fodor and colleagues have demonstrated that individuals high in nPower respond with increased corrugator activation when confronted with dominant others and with decreased activation when dealing with non-dominant interaction partners (Fodor et al., 2006, 2012; Fodor and Wick, 2009). Other studies have used subjective ratings of hedonic well-being to show that nPower predicts individuals' emotional well-being in response to success and failure in the everyday pursuit of power goals (Brunstein et al., 1998; Schultheiss et al., 2008a).

Research on autonomic responses to power incentives shows that nPower predicts distinct hormonal release patterns to dominance and defeat. Men high in nPower respond to a victory in a one-on-one competition against another man with an increase in testosterone, whereas they respond to a defeat with a decrease in this hormone (Schultheiss and Rohde, 2002; Schultheiss et al., 2005b). Women high in nPower show a parallel response pattern to victory and defeat in their estradiol levels (Stanton and Schultheiss, 2007). Power-motivated men and women both respond with increased adrenal catecholamines to power-arousing situations (McClelland et al., 1980, 1985) and with increased cortisol to defeat in such situations (Wirth et al., 2006). These studies suggest that the hypothalamus, a key interface between motivation, endocrine regulation, and behavior (Iversen et al., 2000), is involved in nPower (see Schultheiss, 2013, for a review) and that nPower thus has many of the hallmarks of power/dominance motivation as studied by biopsychologists and neuroscientists (e.g., Sapolsky, 1987; Albert et al., 1992; Johnson et al., 2012).

More evidence that nPower is associated with core motivational processes comes from a brain imaging study in which Schultheiss et al. (2008b) used an oddball detection task to test effects of facial expressions of emotion (FEE) on activation of brain areas that are critically involved in motivational regulation of behavior (striatum, amygdala, insula, orbitofrontal cortex). This work was based on the notion that FEEs represent interpersonal incentives whose reward and punishment value depends both on the emotion displayed by the sender and the motivational needs of the perceiver (Stanton et al., 2010). More specifically, Schultheiss et al. (2008b) expected that for high-power individuals, but not for low-power individuals, angry expressions signal high dominance and thus represent an aversive stimulus and that surprised expressions signal low dominance and thus represent a rewarding stimulus. Except for the amygdala, in which the signal was in the expected direction but too weak to pass a stringent significance threshold (see Hall et al., 2010), individual variations of nPower predicted enhanced brain activation responses in all motivational-brain areas to angry expressions, relative to neutral expressions, and to a lesser extent also to surprised expressions. Notably, nPower-dependent activation increases to dominance-related FEEs were strong and extensive in the dorsoanterior striatum, particularly the caudate head, a key structure for reinforcement learning (Delgado, 2007). This observation plays a key role in our explanation of phenomena associated with nPower-dependent implicit learning, an issue to which we turn next.

## nPower-dependent implicit learning

Implicit learning occurs when a person picks up a regularity in the patterning of environmental cues and uses it to increase response efficiency, above and beyond performance changes unrelated to learning and without being able to explicitly state the regularity (Reber, 1989; Berry, 1996). Although implicit learning is a phenomenon usually studied from the perspective of cognitive psychology, some researchers have extended its range of validity to the social domain (e.g., Lewicki et al., 1989). Lieberman (2000) in particular argued that implicit learning is the basis of social intuition, that is, complex, yet largely automatic behavioral adjustments in response to social feedback that individuals need to make to succeed in their interactions with others.

This social-adjustment view of implicit learning also guided a series of studies we and our collaborators conducted on nPower-moderated responses to dominance contest outcomes. The research was based on the hypothesis that because individual differences in nPower determine to what extent dominating another person is rewarding or being dominated by another person is aversive, implicit learning of behavior that results in these situational outcomes should likewise depend on individual differences in nPower. For instance, because a person high in nPower can enjoy beating an opponent in a direct competition, this person should also better learn whatever he or she has done during the competition to be victorious. In contrast, a person low in nPower should not enjoy a victory against a competitor and therefore also fail to get reinforced for whatever behavior has led to this outcome.

We have tested this hypothesis in a series of studies that combined a dominance-contest paradigm, in which the outcome (victory, defeat) was experimentally manipulated, with implicit-learning tasks that participants worked on during the contest. In all studies, gains in implicit learning were assessed after the contest, or, in the parlance of learning psychology, during extinction, when reinforcement (beating the opponent; being beaten by the opponent) was no longer provided. In all studies, explicit awareness of learning was assessed at the end of data collection, and participants generally had no declarative knowledge of the stimulus-response pattern they had learned. Moreover, when those few participants who showed explicit knowledge of the pattern were excluded from analyses, the results reported in the following remained unchanged, suggesting that explicit awareness of the pattern was not critical for its acquisition and execution.

Using a paper-and-pencil task that allowed participants to learn a repeating pattern of connections between successive numbers, Schultheiss and Rohde (2002) found in a study with male German participants that nPower significantly predicted better learning among contest winners, and worse learning among contest losers, who were also low in activity inhibition, a measure of brain lateralization during stress (Schultheiss et al., 2009). Schiepe-Tiska (2012), who used a computer-administered variant of Nissen and Bullemer's (1987) serial-response-task (SRT) paradigm for the assessment of implicit learning in a similar contest paradigm, recently replicated these results in another study with male German participants. Like Schultheiss and Rohde (2002), Schiepe-Tiska found a joint effect of nPower and contest outcome on implicit learning among participants low in activity inhibition, with nPower predicting better learning among winners, but not among losers.

These findings were replicated and extended to both genders by Schultheiss et al. (2005b) in two studies with US students using the SRT paradigm for the assessment of implicit learning. In these studies, nPower predicted better learning among winners and worse learning among losers in men and women alike and regardless of participants' activity inhibition levels. The findings reported by Schultheiss and Rohde (2002), Schiepe-Tiska (2012), and Schultheiss et al. (2005b) are all consistent with the notion that winners should learn and utilize the fixed sequence inherent in implicit learning tasks only to the extent that they experience the outcome as rewarding (victory) or punishing (defeat), which in turn depends on participants' nPower.

Going beyond the dominance-contest paradigm, Schultheiss et al. (2005a) tested whether individual differences in nPower also predict implicit learning when the action-contingent outcome is the presentation of an FEE. Like Schultheiss et al. (2008b), these authors argued that facial expressions of anger, joy, surprise, and neutrality can be aligned on a dominance dimension, with anger and joy signaling someone else's high dominance and thus being aversive for a power-motivated perceiver, surprise signaling someone else's low dominance, and thus being rewarding for a power-motivated perceiver, and neutrality representing a mid-point on the dominance dimension. Schultheiss et al. (2005a) tested the validity of this proposition by having each of their participants learn three distinct SRT sequences. One sequence was always followed by an emotional face, one always by a neutral face, and one always by no reinforcing stimulus. Emotion (anger, surprise, joy) was varied between subjects. Learning was tested in extinction, that is, when SRT fixed-sequence execution was no longer reinforced by faces. Results showed that compared to learning on neutral-face or no-face sequences, nPower predicted enhanced learning of surprise-face SRT sequences and impaired learning of joy-face sequences. For participants in the angry-face condition, nPower predicted impaired implicit learning overall. These findings suggest that, as proposed by Lieberman (2000), implicit learning is indeed sensitive to social signals such as brief emotional expressions. But like the contest studies, it shows that the meaning of social dominance signals and dominance-related outcomes as hedonically charged rewards and punishers depends on individuals' nPower.

One surprising but very consistent finding in the studies using the SRT paradigm by Schultheiss et al. (2005a,b) and Schiepe-Tiska (2012) was that the effect of nPower on learning emerged for the *accuracy* with which participants executed the fixed sequence (relative to random sequences), but not for a more commonly used indicator of implicit learning, that is, the relative *speed* with which participants executed the fixed sequence. (The task used by Schultheiss and Rohde, 2002, did not allow to differentiate between accuracy and speed effects.) Effects of nPower on speed-based learning emerged only in the FEE-reinforcement study by Schultheiss et al. (2005a). However, these effects were considerably weaker and more dependent on additional factors (e.g., FEE presentation time) than the effects observed for accuracy. Across all studies, the specificity of the effect of nPower on learning accuracy was particularly striking because speed- and accuracy-based indicators of learning were positively correlated (up to *r* = 0.50). How can the differential sensitivity of implicit-learning accuracy and speed for nPower-dependent reinforcement be explained?

## A striatal basis of nPower-dependent implicit learning

We propose that insights from more than a decade of research on the role of the dorsoanterior striatum in early sequence learning, action-outcome learning, and the modulation of learning by dopamine (DA) input to the striatum may help answer this question. Using a serial-response task that could be adapted for use with both primates and human research participants, Hikosaka and colleagues demonstrated, by transient blockade of learning through transmitter antagonists (Miyachi et al., 1997) and by augmentation of learning through electrical stimulation of neuron populations (Nakamura and Hikosaka, 2006; see also Williams and Eskandar, 2006), that the anterior portion of the caudate nucleus is critically involved in the *implicit learning of new visuomotor sequences*, and that such learning is reflected by an increase in sequence execution *accuracy*. In contrast, experimental manipulation of neuronal activity in more posterior parts of the striatum specifically altered the performance of *well-learned sequences* and was reflected in changes in sequence execution *speed* (Miyachi et al., 1997, 2002).

Applied to the previously reviewed findings relating nPower and implicit learning accuracy, this suggests that nPower-dependent modulation of instrumental learning occurs early, during the acquisition of action-outcome contingencies, and is mediated by the dorsoanterior striatum. Such an interpretation would be consistent with the observation of nPower-dependent activation of the caudate head in response to perceived dominance signals (Schultheiss et al., 2008b; Hall et al., 2010), which may have reflected a process related to the recruitment of suitable responses for dealing with the emotional stimulus. It would also be consistent with a hypothesis presented by Hikosaka and colleagues (Hikosaka et al., 1999; Balleine et al., 2007; see also Balleine and O'Doherty, 2010), who have argued that the acquisition of stimulus-guided behavioral sequences in the dorsoanterior striatum, and particularly the head of the caudate, is a form of action-outcome contingency learning that depends on the motivational value of the outcome at the time of acquisition: the higher the reward value of the outcome, the steeper the learning (see, for instance, Delgado et al., 2003). Moreover, Balleine et al. (2007) point out that action-outcome learning mediated by the dorsoanterior striatum is particularly likely to be observed in tasks that have a strong social-interaction component, such as punishing others for transgressing a social norm (de Quervain et al., 2004) or learning to trust another person in an economic exchange (King-Casas et al., 2005). This, too, fits the studies on nPower and learning, which featured “strong” social interactions by using actual face-to-face contest situations to make victory and defeat salient (Schultheiss and Rohde, 2002; Schultheiss et al., 2005b; Schiepe-Tiska, 2012).

Our interpretation of nPower-dependent implicit learning also fits well with Lieberman's (2000) neurocognitive model of social intuition. Like Balleine et al. (2007), Lieberman (2000) argues that intuition based on implicit learning of socially adaptive behavior depends critically on the striatum—effective and sophisticated adaptation of social behavior is possible only to the extent that an intact striatum supports implicit learning processes. Frequently, power-motivated individuals are socially successful not because they try to have impact on others through blunt dominance and aggression—a strategy that is prone to backfire—but by picking up on “behaviors that work,” such as appearing competent and intelligent to others (Schultheiss and Brunstein, 2002), being perceived as charismatic (De Hoogh et al., 2005), or even learning to execute an arbitrary sequence of key presses, as in our contest studies. We argue that the diverse range of sophisticated behaviors that power-motivated individuals learn to employ in their quest for impact depend on striatum-mediated implicit learning that gives rise to such intuitive and successful behavioral strategies. Following Lieberman's (2000) lead, we would therefore predict that a loss of a functional dorsoanterior striatum would equal a loss of sophisticated pursuit of power-related incentives in power-motivated individuals. This is illustrated by a case study of a young woman with bilateral damage of the caudate head, reported by Richfield et al. (1987). Before the damage, the woman had graduated from high school with high honors, held a job, and was happily engaged. Although the woman did not complete a measure on nPower, one can surmise that she expressed whatever degree of nPower she had in well-adjusted ways. After the damage, however, her behavior became socially inappropriate and included vulgarity and violent outbursts, which can be recognized as the prototypical, unsocialized forms of power seeking typically observed in young children (see McClelland and Pilon, 1983).

**Figure 1:**
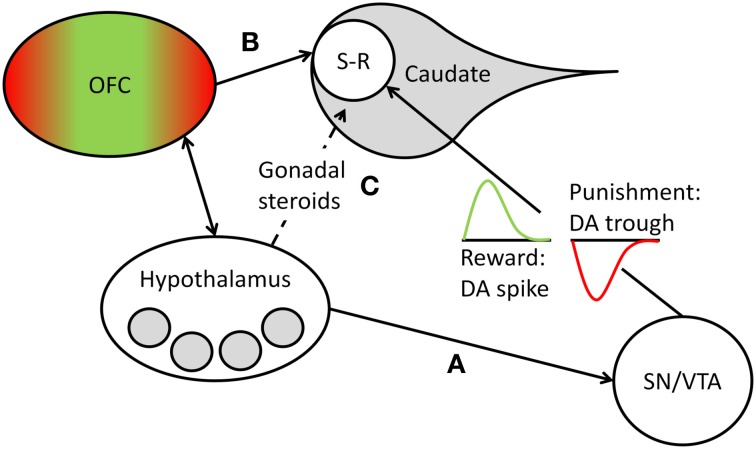
**Overview of the structures, pathways, and processes postulated to mediate nPower effects on implicit learning.** Learning of new stimulus-response (S-R) sequences takes place in the head of the caudate nucleus and is reflected in the accuracy with which S-R sequences are executed (Hikosaka et al., 1999). The motivational value of the outcome of such S-R sequences is encoded by phasic DA release, with a transient spike in DA cell firing marking a reward and leading to reinforcement of the sequence and a transient trough marking a punishment and leading to a suppression of the sequence (see Bromberg-Martin et al., 2010). We propose that in dominance-related contexts, the magnitude of the phasic DA changes that drive S-R learning in the caudate depends on nPower-associated liking of power-specific rewards and disliking of power-specific punishers, with higher nPower leading to greater DA changes in response to such events. We furthermore propose that nPower-dependent scaling of (dis)liking responses to power-specific (dis)incentives takes place in need-specific areas (shaded gray circles) of the hypothalamus and in reward- and punishment-related hedonic evaluation areas of the orbitofrontal cortex (OFC; green represents the medial reward-related areas, red the lateral punishment-related areas; see Kringelbach, 2005), which closely interacts with the hypothalamus. nPower-specific incentive evaluation in these areas can influence striatal S-R learning by **(A)** hypothalamic modulation of DA release from the substantia nigra/ventral tegmental area (SN/VTA), **(B)** direct projections from the OFC to the head of the caudate, or **(C)** indirect modulation of striatal DA release by the influence of nPower on gonadal steroids (estradiol, testosterone), whose levels are regulated by the hypothalamus.

Learning of stimulus-response contingencies in the striatum depends on the release of DA by projections of cells located in the brainstem (substantia nigra [SN] and ventral tegmental area [VTA]). Animal and human studies of implicit sequence learning^1^ show that experimental enhancement and inhibition of DA release lead to corresponding enhancements and impairments of sequence learning (Kumari et al., 1997; Miyachi et al., 1997; Dunnett et al., 2012; for a review, see Udden et al., 2010). Moreover, human research participants show increased DA release in the striatum, including the caudate head, during implicit learning on the SRT (Badgaiyan et al., 2007). Individuals suffering from Parkinson's disease, which is associated with reduced DA levels, show worse implicit sequence learning performance than healthy control participants (Smith and McDowall, 2004). While these studies suggest that the availability of DA at the synapse is a critical requirement for implicit learning to occur, other research, reviewed in Bromberg-Martin et al. (2010), shows more specifically that phasic bursts of DA in the striatum drive action-outcome learning, depending on the motivational value of the outcome generated by the response. Some DA neurons code for the rewarding consequences of an action, marking the event with a brief increase (i.e., spike) of DA release at striatal synapses, whereas other DA neurons code for punishment, as reflected by a brief reduction (i.e., trough) of DA at striatal synapses (Matsumoto and Hikosaka, 2009). If the outcome has no positive or negative motivational value, DA release is neither increased nor reduced. Thus, at the synaptic level, phasic DA changes reinforce action-outcome learning in the case of reward or suppress it in the case of punishment (see Bromberg-Martin et al., 2010). We suggest that in the context of power-relevant person-environment transactions, nPower determines the magnitude of phasic DA release changes in response to action outcomes, because it determines the motivational value of success or failure at having impact on others. Thus, in the dominance contest studies reviewed above, we would have expected high-power individuals, but not low-power individuals, to show marked DA spikes in the dorsoanterior striatum in response to winning a round on a dominance contest. These DA spikes could in turn have reinforced the stimulus-response contingencies inherent in the implicit visuomotor learning task the contest was based on. Conversely, we would have expected high-power individuals, but not low-power individuals, to show marked DA troughs in the dorsoanterior striatum in response to losing a round. These DA troughs could in turn have suppressed the acquisition of stimulus-response contingencies in the learning task (see Figure 1). We propose that this represents the neurophysiological mechanism by which nPower, in interaction with dominance-related rewards and punishments, drives implicit learning in power-relevant situational contexts.^2^

## A broader perspective on the role of the dorsoanterior striatum in implicit motivation

In closing, we want to briefly address the question of where in the brain nPower-associated motivational valuation of an action outcome is encoded and also discuss the broader implications of the model for other implicit motivational needs. Although we have argued that the magnitude of phasic changes in striatal DA release in response to dominance incentives and disincentives reflects nPower-dependent valuation of action outcomes, we do not want to suggest that they represent nPower-dependent neuronal representations of reward evaluation (i.e., liking) or that *all* phasic variations in dopaminergic neurotransmission are driven by nPower. For one, DA responses have been shown to dissociate from liking responses to rewards and punishers and to become associated with incentive-*predicting* cues over time and with the monitoring of prediction accuracy (Schultz, 1998; Bromberg-Martin et al., 2010). Moreover, DA release in the striatum is involved in striving for many different types of incentives, including food, sex, and money, and thus represents a common currency of motivational valuation, not a process specifically linked to one motive, such as nPower. However, DA neurons in the SN and VTA receive inputs from other brain areas that may represent more specific motivational needs and need-specific outcome evaluations and thus may drive DA release in the striatum via their projections to the SN/VTA area. One brain site with particularly extensive projections to this area is the hypothalamus (Gonzalez et al., 2012), which represents physiological and social needs in a domain-specific manner in distinct nuclei (see Schultheiss, 2013) and, as we have pointed out previously, is involved in the nPower-associated release of testosterone in men and estradiol in women. The hypothalamus may also transmit to the SN/VTA domain-specific and topographically distinct hedonic liking signals encoded by the orbitofrontal cortex (OFC; see Kringelbach, 2005), with which it has extensive reciprocal connections (Öngür and Price, 2000). Schultheiss et al. (2008b) and Hall et al. (2010) have argued that these brain sites are particularly likely candidates for representing individual differences in liking responses to motive-specific rewards and punishments, and we suggest that these specific liking responses to power-related rewards and punishments drive responses of DA neurons in the SN/VTA.

It is also conceivable that nPower-specific outcome evaluations influence striatal functions more directly by, for instance, direct projections from the OFC to the dorsoanterior striatum (Öngür and Price, 2000), which may modulate synaptic learning driven by phasic DA changes in a specific manner, or by the effects of nPower-associated testosterone and estradiol, which broadly augment striatal DA effects (e.g., Becker and Rudick, 1999; Frye et al., 2002). The latter suggestion is consistent with the observation by both Schultheiss and Rohde (2002) and Schultheiss et al. (2005b) that in male contest winners and losers, effects of nPower on implicit learning were mediated by changes in testosterone.

Both the notion that need-specific outcome evaluations take place elsewhere in the brain and the fact that the striatum is active during the pursuit of many different motivational incentives suggest that the dorsoanterior striatum, and DA-based learning happening there, may also play a role in other implicit motives, such as the needs for achievement (nAchievement; Pang, 2010) and affiliation (nAffiliation; Weinberger et al., 2010). In support of this notion, Bäumler (1975; reviewed in Schultheiss and Brunstein, 2005) has shown that experimental pharmacological manipulation of DA levels effects changes in a measure of nAchievement, with DA agonists leading to an increase and DA antagonists leading to a decrease of achievement imagery in the stories that research participants write about picture cues related to achievement. Moreover, Hall et al. (2010) report that nAchievement assessed with a picture-story test positively predicts activation of the caudate nucleus in response to anger FEEs in an fMRI study. This observation supports the notion that the striatum plays a role in other implicit motives besides nPower. Interestingly, Hall et al. (2010) also report a *negative* association between nAffiliation and caudate activation in response to angry faces. This suggests that this motive, too, can influence striatal processing of motivational incentives, but perhaps in a different manner than nPower or nAchievement, which were both associated with increased caudate activation in response to anger FEEs (see also Schultheiss et al., 2008b). However, this difference may be due to the fundamentally different meaning of perceived anger expressions as rewards or punishments in the context of power, achievement, or affiliation (see Stanton et al., 2010). Further research is necessary to determine whether nAffiliation, in interaction with positive affiliation-related incentives (e.g., smiling expressions), can also predict increases in anterior striatal activation. Although some evidence already suggests that nAchievement and nAffiliation predict implicit learning that is followed by motive-specific incentives (Schultheiss et al., 2005a; Pang, 2010), more research is also needed to clearly demonstrate when and how these motivational needs influence the implicit acquisition of instrumental behavior. Such evidence would make it appear even likelier that these motives recruit the type of action-outcome-contingency learning associated with the dorsoanterior striatum that we have postulated here.

### Conflict of interest statement

The authors declare that the research was conducted in the absence of any commercial or financial relationships that could be construed as a potential conflict of interest.

## Footnotes

^1^By necessity, sequence learning tasks in animal studies—particularly when rats or mice are used—often differ from those used in human studies. However, because researchers aim to model the animal tasks on the human tasks, they also share key features, such as the association of motor responses with spatially patterned stimuli (e.g., Dunnett et al., 2012). Moreover, when primates are compared to humans, it is even possible to use the same sequence learning tasks (e.g., Hikosaka et al., 2002b).

^2^Other brain areas, such as the amygdala, contribute to emotional processing in general (e.g., Sergerie et al., 2008) and instrumental learning in particular (e.g., Killcross et al., 1997), and DA projections to the amygdala are known to influence emotional-motivational processing (for an overview, see for instance Cardinal et al., 2002). While we think that a broader network of brain areas including the amygdala and other structures is involved in implicit power motivation (see Figure 1; see also Schultheiss et al., 2008b; Hall et al., 2010), we focus selectively on the role of the striatum in nPower-dependent implicit learning in the present paper.
